# Ageing, dementia and society – an epistemological perspective

**DOI:** 10.1186/s40064-015-0910-1

**Published:** 2015-03-20

**Authors:** Klaus Heese

**Affiliations:** Graduate School of Biomedical Science and Engineering, Hanyang University, 222 Wangsimni-ro, Seongdong-gu, Seoul 133-791 Republic of Korea

**Keywords:** Aging, Belief, Dementia, Psychology, Religion, Culture, Society, Education

## Abstract

Recent data show that as populations age, the number of people affected by neurodegenerative dementia is growing at an epidemic pace in various regions of the world. This cross-cultural study examined the relationships among age, gender, ethnicity, religion, and education as well as the attitudes and perceptions related to ageing and dementia. A random sample of 980 participants was selected to represent the multicultural population of Singapore. Data were collected using standardised questionnaires through online portals and by conducting interviews. These data were ultimately analysed by comparing percentage responses and correlation coefficients and by conducting a multiple regression analysis. The results indicate that the perceptions and attitudes of individuals toward ageing and dementia differ among different age groups. Moreover, the level of education attained was significantly correlated with understanding dementia; regardless of education level, Christians had the most positive mindset toward dementia, although most religious individuals did not believe in divine healing. In this study, it was determined that attitudes and perceptions about ageing and dementia are influenced by multiple factors, such as education, age, and religion, and that it is imperative that younger generations develop coping strategies, including healthy lifestyles and social and/or religious communities to provide quality care to the elderly, in general, and to dementia patients, in particular.

## Introduction

It is well known that increasing longevity and declining fertility rates are shifting the age distributions of populations toward older age groups in many parts of the world, including Singapore, Europe, the United States of America (USA) and, in fact, most industrialised nations (Anderson, et al. 2000). The most rapid growth in the elderly population is occurring in China, India, and their South Asian and Western Pacific neighbours. Improved sanitation, medical technology, and healthcare services, as well as increased individual wealth, have all contributed to rising life expectancy (Ministry of Community Development 1999). However, the rate at which a population ages differs substantially from one country to another and is dependent on socioeconomic development (Cheng and Heller 2009). According to the United Nations demographics indicator, the relative population of individuals aged 65 and above will increase rapidly in industrialised countries by an average of 16.8 percent between 2000 and 2020 (Anderson and Hussey 2000). In 2010, this shift accelerated when baby boomers began to turn 65 years of age.

As the population grows older, age-related diseases such as dementia will increase and issues such as providing proper healthcare and disease treatment will come to the forefront. The resulting financial and personal costs might devastate the world's economic and healthcare systems, in addition to burdening many families worldwide. Changes in public policies must be implemented to accommodate financial security, healthcare provision and living arrangements (Chan 2001). Because different cultures address ageing-related issues such as dementia differently, the governments of industrialised countries should seek to increase awareness of the socioeconomic problems associated with ageing-related issues.

Our understanding of dementia (e.g., Alzheimer’s disease (AD)) is limited by the characteristics of the people who have traditionally been included in investigations. There is a need for basic sociological and anthropological data about dementia, AD, families and caregiving in specific cultural, social, and regional contexts to provide a working platform for effective service, education and program delivery. Thus, the pilot study presented here is the first of its kind and is intended to provide insight into the level of awareness of Singaporean residents regarding ageing and dementia, given the various cultures, religions and ethnicities that reside in the nation.

### Dementia

Dementia is a cognitive disorder that affects the brain and results in failing memory and personality changes (Martin 2009). As of 2010, there were an estimated 35.6 million persons with dementia worldwide. This number will nearly double every 20 years, resulting in an estimated 65.7 million in 2030 and 115.4 million in 2050. Much of this increase will occur in developing countries. At present, 58 percent of persons with dementia reside in developing countries; by 2050, this figure will rise to 71 percent. By 2050, individuals aged 60 years and over will account for 22 percent of the world’s population, with four-fifths living in Asia, Latin America and Africa (Alzheimer’s Disease International 2009; Ferri et al. 2005). Europeans are also plagued by mental and neurological illnesses, with nearly 165 million people (38 percent of the population) suffering from disorders such as depression, anxiety, insomnia or dementia each year (Wittchen et al. 2011).

These diseases are not without cost to communities. The total estimated worldwide cost of dementia was US$604 billion in 2010; approximately 70 percent of these costs was from western Europe and North America (Alzheimer’s Disease 2010; Gustavsson et al. 2011).

### Dementia, science, societies, religion, and education

Dementia can be viewed from different perspectives and from scientific, anthropological or socio-cultural frames of reference. From a scientific perspective, scientists continue to research the causes of and target treatments for dementia on a cellular level (Heese and Akatsu 2006; Kumari and Heese 2010). Research scientists in numerous countries have proposed explanatory and causality models for dementia (Jeong et al. 2007; Kleinman 1978; Lynch and Medin 2006; Ravaglia et al. 2007). From a socio-cultural perspective, social scientists and anthropologists study how humans interact with and interpret dementia, which helps to develop treatments from a humanistic perspective (Hinton et al. 1999).

Various societies from across the globe traditionally believed that mental and emotional disorders were caused by malevolent supernatural forces (Gaw 1993). However, with the rise of the psychiatric and neuroscientific disciplines, scientific explanations have taken precedence over supernatural conceptualisations (Landrine and Klonoff 1994; Sheikh and Furnham 2000). In this scientific era, individuals are more reliant on intellectual and scientific explanations than on spiritual or religious leaders to examine their lives. In Singapore, notions involving abstract thinking and being open-minded about and tolerant of different religions is linked to Singaporeans adopting modern values common to developed nations (Chang et al. 2003). In addition, in our educational system, scientific rationality has revolutionised the interpretation of phenomena and altered the epistemological basis for understanding how the world works (Glaeser and Sacerdote 2002). Research has shown that a secular education emphasises secular beliefs that are at odds with traditional religious views, thereby affecting individuals’ beliefs in supernatural phenomena or miracles as cures for mental health disorders (Ellison and Sherkat 1995; Mathews 2010). In the USA, public education has been designed to encourage the adoption of a secular and nationalist belief system (Bowles & Gintis 1976; Chaves 1994; Hadden 1987). More liberal education reform will certainly influence individual’s perspectives of the world around them. Research has also shown that low levels of education can influence the prevalence of dementia across different populations (Katzman 1993; De Ronchi et al. 1998).

### Dementia, aging, and ethnicity

Singapore’s population comprises 74.2 percent Chinese, 13.4 percent Malaysian, 9.2 percent Indian, and 3.2 percent minority ethnic groups (Singapore Department of Statistics 2009). Ethnicity and culture can influence perspectives on and interpretations of ageing and dementia. Different societies have different models that interpret mental disorders and may involve supernatural elements that structure how individuals address such mental and emotional issues (William and Healy 2001). In every society, various ethnic groups perceive various mental illnesses from different cultural frames of reference (Garro 1994). Because of intra- and inter-ethnic diversity across populations, culturally based conceptions may also influence individual perceptions of ageing and dementia (Hinton et al. 1999). Research by Kane and Williams (2000) has shown that individuals who sought healthcare services were often linked to an ethno-cultural identity (e.g., the African-American community seeking clergy for help and counsel in the USA). For example, in the USA, African-Americans underutilise mental health services as a result of cultural mistrust (Whaley 2001). Other studies have shown that cultural differences and culturally based conceptions of disease have led to different patterns in the use of medical assistance (Doescher et al. 2000; Lindström et al. 2001; Wiking et al. 2004). In Europe, research has shown that the Spanish have a more positive attitude toward seeking help for mental health issues than Germans; this outcome may be because Spanish individuals have more unmet needs (ten Have et al. 2010).

### Aging, religion, and health

Caretaking is becoming a major socioeconomic issue for almost all industrialised countries affected by ageing and associated dementia (Agnus and Reeve 2006; National Institute on Ageing (NIA), National Institute of Health (NIH), U.S. Department of Health and Human Services, and World Health Organisation (WHO) (2011)). As a country becomes more industrialised, more individuals regard honouring their traditions and their elders as a socio-cultural anachronism, and thus cultural traditions in general, and honouring elders in particular, are becoming less common (Watkins 2010). In 1961, social gerontologists posited the disengagement theory of ageing, which posited that as individuals age, they disengage from religious and family activities, tendencies that may be detrimental to physical and mental health (Hooyman and Kiyak 1996).

Individuals’ spirituality or religiosity may also affect their perceptions of and attitudes toward preventing or seeking help for mental health diseases (Vogel et al. 2007). Spirituality may be understood as the driving force that confers meaning, stability, and purpose to life through a relationship with dimensions that transcend the self (Oldnall 1996; Reed 1987). Another definition of spirituality involves family support and praying with others as an expression of faith (Rovers and Kocum 2010). Different religions advocate different teachings. Rabinowitz et al. (2010) demonstrated that negative religious coping was significantly associated with increased cumulative health risk. Many health practitioners and caregivers have also recognised the importance of spirituality in fostering positive outcomes related to mental health and quality of life (Sawatzky et al. 2005), including spirituality through religions such as Christianity, Islam, Hinduism, Buddhism, Taoism, and Freethought (Freethought refers to unorthodox beliefs that do not conform to any religious beliefs; practitioners of Freethought are called Freethinkers).

### Attitudes toward and perceptions of aging and dementia

Brain and mind research often concentrate on such topics as conscious and unconscious perception, decision-making, language, and other key determinants of any mental function: brain plasticity and lifespan ontogeny as well as mental disorders and brain dysfunction (Collin and van den Heuvel 2013; Nikolic 2011; Scheithauer et al. 2008). These topics are certainly closely interrelated and potentially embedded in basic and clinical research projects. Empirical research is typically conducted in close connection with conceptual philosophical analysis of key terms such as ‘decision’, ‘free will’ and ‘consciousness’ and with an evaluation of the ethical and anthropological consequences of such research. At present, the rapidly evolving fields of cognitive modelling and computational neuroscience, as well as basic and clinical research, play a key role in the brain and mind research community. Recent technological and scientific progress in the brain sciences now makes it possible to empirically address fundamental questions of the mind sciences. Research on human decision-making, consciousness, language, or game theory – to name just a few specific areas – can benefit from experimental approaches originally developed within the life sciences, in general, and the field of neuroscience, in particular. Conversely, support from the neuropsychological mind sciences is essential to this research on both the conceptual and methodological levels.

However, it is difficult to conceive of how the implications that brain research has for ‘free will’ and ‘consciousness’, for example, can be evaluated in the absence of a coherent philosophical concept of consciousness or free will. Therefore, it is essential to create an institutional setting for intensive cooperation between researchers from the different fields involved. Consequently, in recent years, numerous new research areas have been termed neuro-X, such as neuro-philosophy, neuro-ecology, neuro-ethology, and neuro-economics. While these new areas have created much excitement, they are still in their infancy and await rigorous scientific and conceptual scrutiny.

Thus, all of this interdisciplinary research on topics related to the mind needs to be conceived and derived on the basis of operational definitions that can be mapped onto those definitions used in the brain sciences, that is, we need to develop a new lingua franca for brain and mind research. This process is not only confined to terminological and conceptual translation but also calls for new types of mathematical descriptions and computational models. The rapidly evolving fields of cognitive modelling and computational neuroscience will thus also play a key role in a scientific community that strives to integrate brain and mind research.

Taking the above into account, we must recall that we are addressing human beings that, despite having the same origins and thus the same nature (from an independent, objective point of view), live in different countries, societies, and communities, with different environments, educational backgrounds and knowledge, and in different cultures, all of whom follow various beliefs based on their religion (e.g., Christianity, Islam, Judaism, Buddhism, Taoism, Hinduism, Free Thinker, Others, etc.). These factors certainly affect a human being’s actions and decisions. To overcome this socioeconomic problem, a nation’s leadership must increase awareness of it. Unfortunately, most societies do not recognise the impact of ageing, ageing-related lifestyles, and dementia on their society, and in particular, societies do not recognise that ageing is a major risk factor for dementia (Exalto et al. 2014; Llibre 2013; Manavalan et al. 2013; Negash et al. 2013; Sato and Morishita 2013; Tolppanen et al. 2014). This issue must be addressed to a nation’s society at an early stage – to the younger generations - while taking into account specific cultural, ethnic, and religious backgrounds of individuals, as different sub-communities may have different approaches to these problems.

### Significance and objective – Singapore’s perceptions of aging and dementia

Because ageing and dementia are viewed as major socioeconomic threats to a society (Agnus and Reeve 2006; National Institute on Aging (NIA), National Institute of Health (NIH), U.S. Department of Health and Human Services and World Health Organisation (WHO) 2011), it is imperative to investigate general awareness of ageing-related dementia in a society from an appropriate interdisciplinary perspective, considering a society’s specific ethnic, cultural, and religious background.

**Figure 1:**
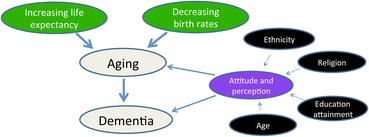
**Increasing longevity and decreasing fertility rates lead to an ageing population and the proliferation of age-related diseases.** Multiple factors affect attitudes toward and perceptions of ageing and dementia.

Thus, as Singapore’s population ages, research on how each of the above-noted factors affects different ethnic groups at the macro level has become increasingly relevant. The approach employed here integrates ideas from multiple disciplines: dementia was examined from an epistemological perspective to determine how ethnicity, religion, age, and educational level influence individuals’ lifestyles and their perceptions of ageing and dementia (Figure 1).

In particular, I attempted to determine whether ethnicity, age and religion influence individuals’ perceptions of ageing and dementia. Participants with different educational levels were studied to determine whether education affected their religious beliefs and influenced their perceptions of ageing and dementia. Finally, a multiple regression analysis was performed to examine how each of these factors — age, religion, education, and ethnicity — affects individual perceptions of ageing and dementia.

## Methods

### Participants

**Figure 2:**
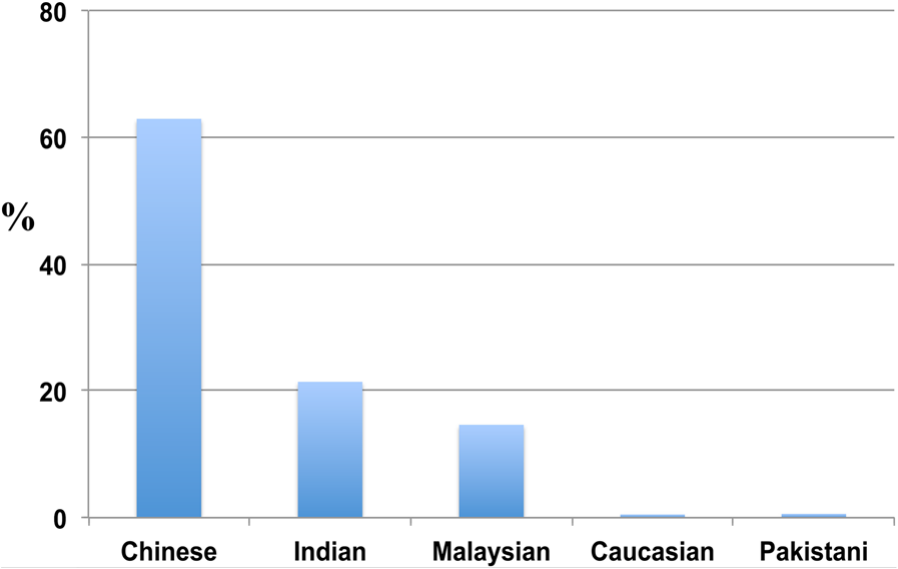
**The distribution of the sample population is categorised into different ethnic groups.** Comprising Chinese (62.9 Percent), Indian (21.4), Malaysian (14.6 Percent), Caucasian (0.5 Percent) and Pakistani (0.6 Percent).

The full random sample was categorized according to ethnicity, age, religion, gender, and education (Table 1). Prior informed consent was obtained from all participants of this study. From the original 1,006 participants, 26 responses were discarded due to incompleteness or erroneous answers. The remaining 980 participants, sampled across the multicultural population of Singapore, comprised the following ethnic groups: 62.9 percent Chinese, 21.4 percent Indian, 14.6 percent Malaysian, 0.5 percent Caucasian, and 0.6 percent Pakistani (Figure 2).

**Table 1:** **Distribution of participants**

			**Ethnic groups**		
**Variables**	**Chinese**	**Malaysian**	**Indian**	**Caucasian** ^**a**^	**Pakistani** ^**b**^
	***n*** **=617 (%)**	***n*** **=143 (%)**	***n*** **=209 (%)**	***n*** **=5 (%)**	***n*** **=6 (%)**
**Age in years (** ***n*** **)**					
19-30 (*n*=655)	438 (71.0)	82 (57.3)	126 (60.3)	3 (60.0)	6 (100.0)
31-40 (*n*=100)	49 (7.9)	19 (13.3)	32 (15.3)	0 (0.0)	0 (0.0)
41-55 (*n*=104)	59 (9.6)	23 (16.1)	21 (10.0)	1 (20.0)	0 (0.0)
56-65 (*n*=106)	59 (9.6)	18 (12.6)	29 (13.9)	0 (0.0)	0 (0.0)
>65 (*n*=15)	12 (1.9)	1 (0.7)	1 (0.5)	1 (20.0)	0 (0.0)
**Religion (** ***n*** **)**					
Christianity (*n*=218)	188 (30.5)	7 (4.9)	19 (9.1)	4 (80.0)	0 (0.0)
Islam (*n*=171)	9 (1.5)	136 (95.1)	20 (9.6)	0 (0.0)	6 (100.0)
Hinduism (*n*=156)	1 (0.2)	0 (0.0)	155 (74.2)	0 (0.0)	0 (0.0)
Taoism (*n*=45)	45 (7.3)	0 (0.0)	0 (0.0)	0 (0.0)	0 (0.0)
Buddhism (*n*=194)	187 (30.3)	0 (0.0)	7 (3.3)	0 (0.0)	0 (0.0)
Freethought (*n*=193)	186 (30.1)	0 (0.0)	6 (2.8)	1 (20.0)	0 (0.0)
Others (*n*=3)	1 (0.2)	0 (0.0)	2 (1.0)	0 (0.0)	0 (0.0)
**Gender (** ***n*** **)**					
Male (*n*=400)	200 (32.4)	65 (45.5)	128 (61.2)	2 (40.0)	4 (67.0)
Female (*n*=580)	417 (67.6)	78 (54.5)	81 (38.8)	3 (60.0)	2 (33.0)
**Marital status (** ***n*** **)**					
Single (*n*=682)	456 (73.9)	86 (60.1)	132 (63.2)	3 (60.0)	5 (83.3)
Married (*n*=298)	161 (26.1)	57 (39.9)	77 (36.8)	2 (40.0)	1 (16.7)
**Education (** ***n*** **)**					
PSLE (*n*=82)	55 (8.9)	15 (10.5)	12 (5.7)	0 (0.0)	0 (0.0)
‘O’ Levels (*n*=106)	62 (10.0)	17 (11.9)	27 (12.4)	0 (0.0)	0 (0.0)
‘A’ Levels (*n*=91)	74 (12.0)	10 (7.0)	7 (3.3)	0 (0.0)	0 (0.0)
ITC/NITEC (*n*=23)	6 (1.0)	9 (6.3)	8 (3.8)	0 (0.0)	0 (0.0)
Diploma (*n*=131)	78 (12.6)	32 (22.3)	21 (9.6)	0 (0.0)	0 (0.0)
Bachelor’s (*n*=189)	115 (18.6)	25 (17.5)	46 (22.0)	2 (40.0)	1 (16.7)
Bachelor’s (with honours) (*n*=301)	215 (34.8)	32 (22.4)	50 (23.9)	1 (20.0)	3 (49.9)
Master’s (*n*=40)	8 (1.3)	2 (1.4)	28 (13.4)	1 (20.0)	1 (16.7)
MD (*n*=1)	0 (0.0)	0 (0.0)	1 (0.5)	0 (0.0)	0 (0.0)
PhD (*n*=13)	3 (0.5)	0 (0.0)	9 (4.3)	0 (0.0)	1 (16.7)
Others (*n*=3)	1 (0.2)	1 (0.7)	0 (0.0)	1 (20.0)	0 (0.0)

^a,b^Results pertaining to these groups should be interpreted with caution because the sample size is small.

The educational attainment of the respondents was quantitatively analysed by comparing the percentage of responses to questions related to dementia. The PSLE (primary education), O (secondary education), A (high school education), ITC/NITEC (technical education) and Diploma (polytechnic education) levels were categorised into ‘Diploma holders and below’ because the difference between each of them was not significant. To facilitate comparison and analysis, educational levels were ultimately categorised into ‘Diploma and below’, ‘Undergraduate degree’ and ‘Postgraduate degree’.

**Figure 3:**
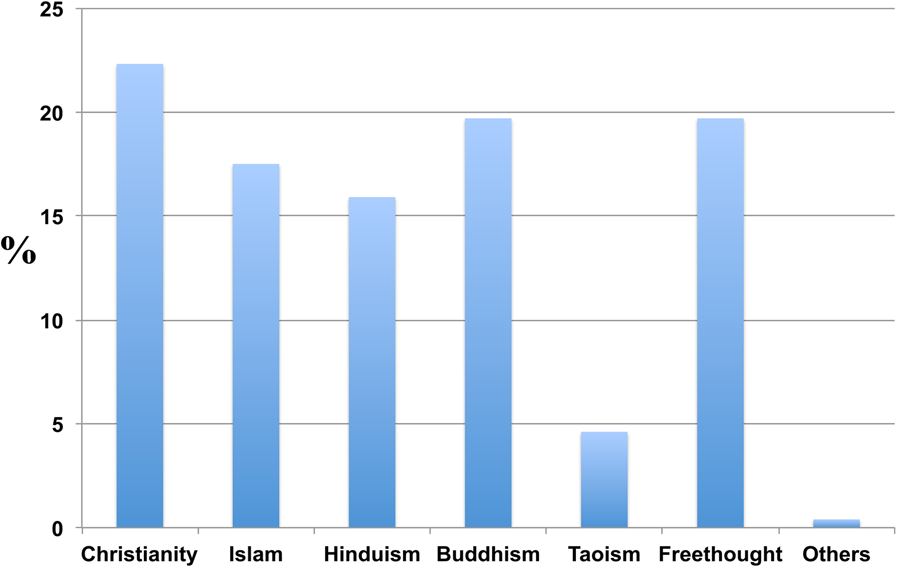
**Random sample population of respondents from different religious backgrounds.** They included Christianity (22.3 Percent), Islam (17.5 Percent), Hinduism (15.9 Percent), Buddhism (19.7 Percent), Freethought (19.6 Percent), Taoism (4.6 Percent), and Others (0.4 Percent).

The participants were categorised into different age groups (66.8 percent were between 19 and 30 years old (Table 1)) and different religious groups. Participants’ religious backgrounds included Christianity, Islam, Hinduism, Buddhism, Freethought, Taoism, and others (Figure 3).

**Figure 4:**
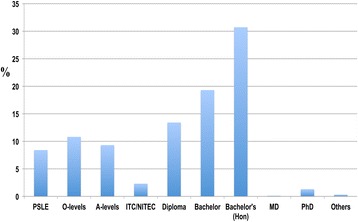
**The educational backgrounds of respondents.** They included PSLE (8.4 percent), ‘O’ levels (10.8 percent), ‘A’ levels (9.3 percent), ITC/NITEC (2.3 percent), Diploma (13.4 percent), Bachelor’s (19.3 percent), Bachelor’s (honours) (30.7 percent), Master’s (4.1 percent), MD (0.1 percent), PhD (1.3 percent) and others (0.3 percent).

The respondents’ levels of educational attainment included primary education (PSLE, Primary School Leaving Examination), secondary education (‘O’ levels), technical education (ITC/NITEC, Industrial Technician Certificate/National Institute of Technical Education Certificate), polytechnic education (Diploma), and tertiary education (Bachelor’s, Bachelor’s (with honours), Medical Degree (MD), Master’s, and PhD) (Figure 4).

### Procedure

Standardised questionnaires (administered online, as return letters, or by personal interview) were conducted across Singapore to assess the perceptions of Singapore’s population regarding ageing and dementia and included a 5-point Likert scale with responses attached to each question ranging from 1 (strongly disagree) to 5 (strongly agree) (Sheikh and Furnham 2000). The questionnaires included questions about respondents’ knowledge of dementia (e.g., its symptoms), their knowledge of the causes of dementia, their general awareness of dementia, and the possibility that ageing could be a major risk factor for dementia. They were also asked their opinions regarding the potential influence of their lifestyle, beliefs and religious practices on ageing and dementia and their attitudes toward placing dementia patients in specialised care facilities or homes. The inquiry included questions on the social implications of ageing for society and for the country’s healthcare system. For ease of comparison, Likert subscales of ‘strongly disagree’ and ‘disagree’ were combined into a single subscale of ‘disagree’; likewise, ‘strongly agree’ and ‘agree’ were combined into an ‘agree’ subscale (3-point Likert scale). A comparative analysis was conducted across ethnic groups to determine any difference in their lifestyles and perceptions of ageing and dementia. Within each ethnic group, participants from different age groups and educational and religious backgrounds were analysed to identify a relationship between their backgrounds and responses. Intra- and inter-ethnic group analyses were also performed for the Singaporean population.

### Data analysis

The relationships among age, gender, ethnicity, religion, and educational attainment on attitudes toward ageing and dementia were ultimately analysed by comparing percentage responses, using correlation coefficients and performing a multiple regression analysis. Religion and educational levels were examined to determine whether educational attainment affected religious beliefs and how it might affect coping strategies. A multiple regression analysis was performed to test this hypothesis and determine how various variables (age, gender, ethnicity, religion, and educational attainment), when taken together, would affect perceptions of and attitudes toward coping with ageing and dementia.

## Results

### Attitudes and perceptions of different ethnic groups regarding ageing and dementia

The responses of Chinese, Malaysian, Indian, and other ethnic groups were tabulated and compiled (Table 2).

**Table 2:** **Perceptions of different ethnic groups toward ageing and dementia**

		**Ethnic groups**			
	**Chinese**	**Malaysian**	**Indian**	**Caucasian** ^**a**^	**Pakistani** ^**b**^
	***n*** **=617**	***n*** **=143**	***n*** **=209**	***n*** **=5**	***n*** **=6**
	**%**	**%**	**%**	**%**	**%**
**Have heard of dementia**					
Completely unaware of it	5.5	11.2	12.0	20.0	16.7
May have heard of it	5.2	5.6	6.7	0.0	33.3
Not sure	18.5	11.9	9.6	0.0	0.0
Somewhat aware of it	51.5	36.4	43.5	80.0	16.7
Completely aware of it	19.3	35.0	28.2	0.0	33.3
**Ageing is a major risk factor for dementia**					
Strongly disagree	4.1	7.7	4.8	40.0	0.0
Disagree	11.5	15.4	12.4	40.0	33.3
Not Sure	37.1	21.7	26.8	0.0	33.3
Agree	35.3	38.5	34.9	20.0	0.0
Strongly agree	12.0	16.8	21.1	0.0	33.3
**People with dementia should be placed in homes**					
Strongly disagree	22.9	25.9	25.8	40.0	50.0
Disagree	47.0	41.3	42.1	20.0	33.3
Not Sure	18.3	18.2	15.8	40.0	16.7
Agree	6.5	9.1	11.0	0.0	0.0
Strongly agree	5.3	5.6	5.3	0.0	0.0
**People with severe dementia should be cared for by professionals instead of family members**					
Strongly disagree	8.3	14.0	11.0	0.0	16.7
Disagree	35.2	32.9	23.9	40.0	16.7
Not sure	27.1	16.8	18.2	40.0	49.9
Agree	21.7	26.6	32.1	20.0	16.7
Strongly agree	7.8	9.8	14.8	0.0	0.0
**Healthy lifestyle reduces the occurrence of dementia**					
Strongly disagree	2.6	5.6	1.4	20.0	0.0
Disagree	20.1	12.6	9.6	40.0	16.7
Not Sure	34.5	21.0	22.0	20.0	0.0
Agree	30.0	39.2	39.2	20.0	50.0
Strongly agree	12.8	21.7	27.8	0.0	33.3

^a,b^Results for these groups should be interpreted with caution, as the sample size is small.

**Figure 5:**
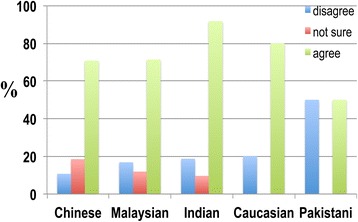
**The responses of participants to the following statement: ‘I have heard of dementia’.** The responses of participants from each of the ethnic groups were rearranged into a 3-point Likert subscale format of ‘disagree’, ‘not sure’ and ‘agree’. At least 70.8 percent of Chinese, 71.4 percent of Malaysians, 71.7 percent of Indians, 80.0 percent of Caucasians, and 50.0 percent of Pakistanis had heard of dementia, which suggests that the majority of the Singaporean population was aware of dementia.

The results reveal that the majority of the Singaporean population was aware of dementia (Figure 5).

**Figure 6:**
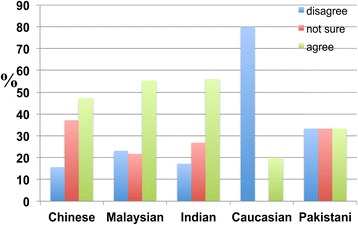
**Responses of participants to the following statement: ‘Ageing is a major risk factor for dementia’.** When asked whether ageing is a major risk factor for dementia, no significant difference was observed between the responses of the ethnic groups. At least 47.3 percent of Chinese, 55.3 percent of Malaysian, 56.0 percent of Indians, 20.0 percent of Caucasians, and 33.3 percent of Pakistanis agreed that ageing is a major risk factor for dementia, whereas 15.6 percent of Chinese, 23.1 percent of Malaysians, 17.2 percent of Indians, 80.0 percent of Caucasians, and 33.3 percent of Pakistanis disagreed.

However, when asked whether ageing is a major risk factor for dementia, no obvious difference was observed between the responses of each ethnic group (Figure 6).

Approximately 70 percent of respondents from the Chinese, Indian, and Malaysian ethnic groups were aware of dementia, but only approximately 50 percent of them believed that ageing was a major risk factor for dementia.

**Figure 7:**
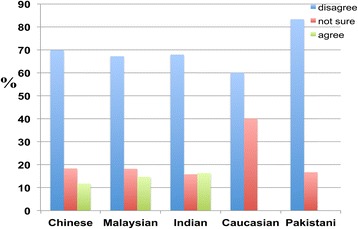
**Participant responses regarding whether dementia patients should be placed in caretaking centres (homes).** Participants were categorised according to their ethnic groups. Significant differences in the responses of participants were observed, with 69.9 percent of Chinese, 67.2 percent of Malaysians, 67.9 percent of Indians, 60.0 percent of Caucasians, and 83.3 percent of Pakistanis disagreeing with placing dementia patients in homes, whereas 11.8 percent of Chinese, 14.7 percent of Malaysians, 16.3 percent of Indians, 0.0 percent of Caucasians, and 0 percent of Pakistanis agreed with placing dementia patients in homes.

A clear difference in participant response was observed with respect to opinions about placing dementia patients in specialised care facilities or homes. Approximately 70 percent of respondents from all ethnic groups disagreed with the notion of placing elderly patients with dementia in specialised care-taking centres (homes). While the majority disagreed with placing dementia patients in specialised care-taking centres, a significant number remained uncertain across all ethnic groups (Figure 7).

**Figure 8:**
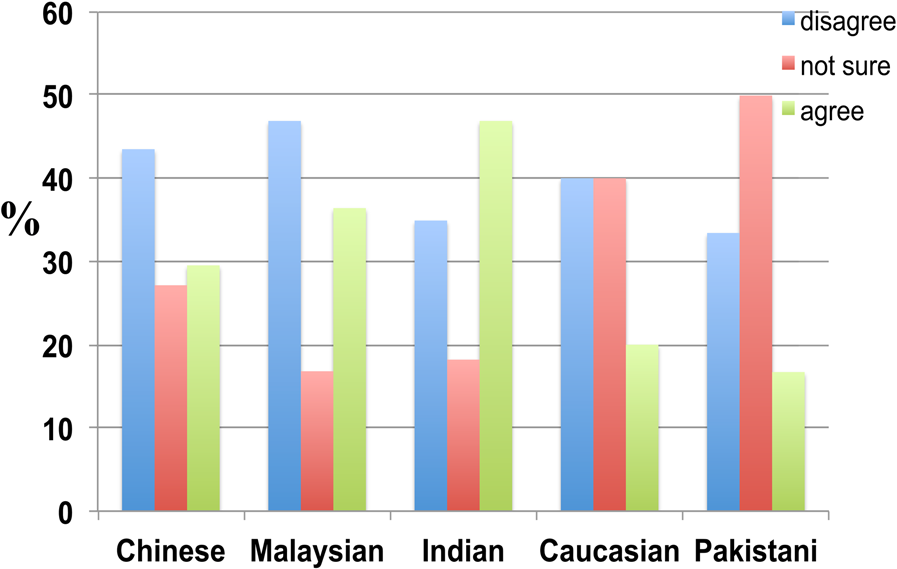
**The responses of participants from different ethnic groups to the following statement: ‘Dementia patients should be taken care of by professionals instead of family members’.** When asked whether professionals should be employed to care for dementia patients, there was no significant difference in responses across ethnic groups, with 43.5 percent of Chinese, 46.8 percent of Malaysians, 34.9 percent of Indians, 40.0 percent of Caucasians, and 33.4 percent of Pakistanis who disagreed with hiring professionals to care for dementia patients to some extent (instead of using family members) and 29.4 percent of Chinese, 36.4 percent of Malaysians, 46.9 percent of Indians, 20.0 percent of Caucasians, and 16.7 percent of Pakistanis who at agreed with the same proposition to some extent.

When asked whether professionals should be employed to care for dementia patients, a higher percentage of participants from the Chinese and Malaysian ethnic groups clearly disagreed with hiring professionals (instead of using family members) than agreed. However, in the Indian ethnic group, more participants agreed with hiring professionals to care for dementia patients than those who disagreed (Figure 8).

**Figure 9:**
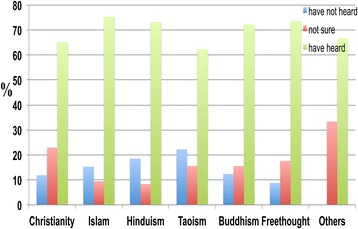
**The responses of participants from different ethnic groups to the following statement: ‘A healthy lifestyle reduces the occurrence of dementia’.** With respect to the effect of a healthy lifestyle on dementia, 42.8 percent of Chinese, 60.9 percent of Malaysians, 67.0 percent of Indians, 20.0 percent of Caucasians, and 83.3 percent of Pakistanis agreed that it reduces or prevents the occurrence of dementia. A higher percentage of Malaysians and Indians than Chinese thought that a healthy lifestyle reduces the occurrence of dementia. Of Caucasians, 60.0 percent disagreed with the proposition that a healthy lifestyle reduces or prevents dementia (which should be interpreted with caution, as the sample size is small).

With respect to the effect of a healthy lifestyle on dementia, a higher percentage of Malaysians and Indians than Chinese thought that a healthy lifestyle reduces the occurrence of dementia (Figure 9).

### Attitudes and perceptions from different religious backgrounds regarding ageing and dementia

**Figure 10:**
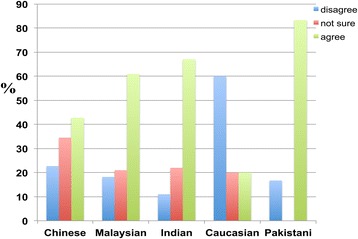
**The response distribution of participants from different religious backgrounds to the following statement: ‘I have heard of dementia’.** Participants had different religious backgrounds, including Christianity, Islam, Hinduism, Buddhism, Freethought, Taoism, and others. A high percentage of participants from all religions were aware of dementia.

The majority of individuals across all religions were aware of dementia (Figure 10).

**Figure 11:**
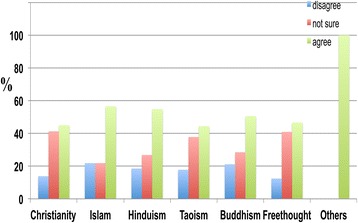
**The responses of participants from different religious groups to the following statement: ‘Ageing is a major risk factor for dementia’.** Christians (45.0 percent), Muslims (56.5 percent), Hindus (54.8 percent), Buddhists (50.5 percent), Freethinkers (46.6 percent), Taoists (44.4 percent), and others (100.0 percent) thought that ageing was a major risk factor for dementia (Figure 10), whereas other Christians (13.8 percent), Muslims (21.8 percent), Hindus (18.5 percent), Buddhists (21.1 percent), Freethinkers (12.4 percent), Taoists (17.8 percent), and others (0.0 percent) disagreed.

Although individuals across all religious groups agreed that ageing was a major risk factor for dementia, approximately 50 percent were either uncertain or even disagreed (Figure 11).

All religions exhibited a higher percentage of participants who thought ageing was a major risk factor for dementia, and there was no significant difference in the responses of participants who thought that ageing was not a major risk factor for dementia.

**Figure 12:**
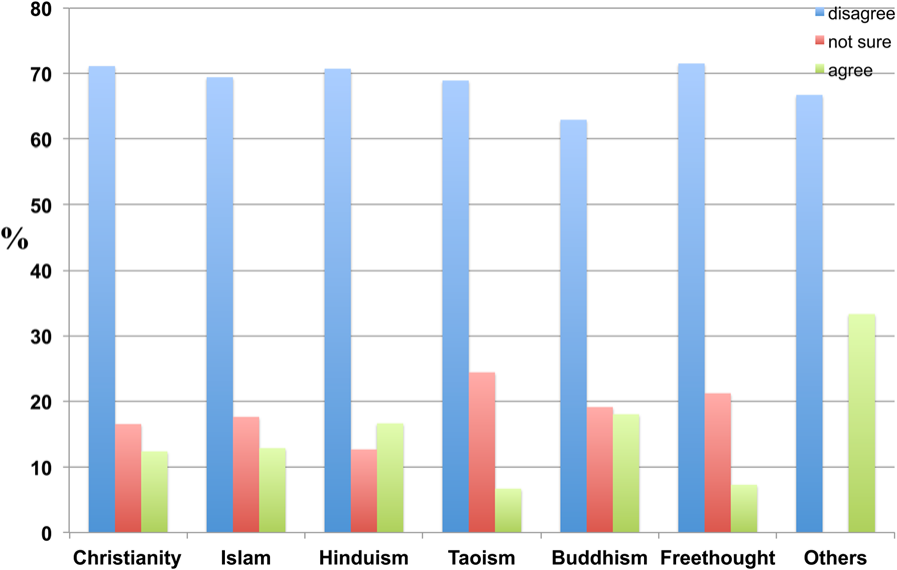
**The responses of participants from different religious groups to the following statement: ‘Dementia patients should be placed in homes’.** Although higher percentages disagreed, other Christians (12.4 percent), Muslims (12.9 percent), Hindus (16.6 percent), Buddhists (18.0 percent), Freethinkers (7.3 percent), Taoists (6.7 percent) and others (33.3 percent) agreed with the practice.

Across religious groups, there was an obvious difference in the percentage of individuals who disagreed and agreed that dementia patients should be placed in homes (Figure 12).

**Figure 13:**
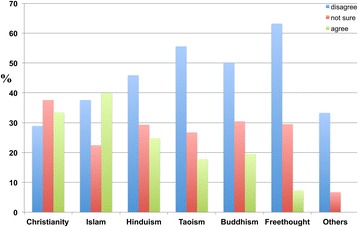
**The responses of participants from various religious groups to the following statement: ‘Having a religion alleviates the effects of dementia’.** Christians (33.5 percent), Muslims (40.0 percent), Hindus (24.8 percent), Taoists (17.8 percent), Buddhists (19.6 percent), Freethinkers (7.3 percent) and others (0.0 percent) agreed that religion helps to alleviate the effects of dementia. However, the remaining Christians (28.9 percent), Muslims (37.6 percent), Hindus (45.9 percent), Taoists (55.6 percent), Buddhists (50.0 percent), Freethinkers (63.2 percent) and others (33.3 percent) disagreed.

While the responses were nearly equal among Christian, Muslim, and Hindu respondents, Freethinkers (63.2 percent) and Taoists disagreed with the proposition that religion helps to alleviate the effects of dementia. In addition, there was a significant difference between the percentage of Freethinkers who agreed and disagreed that religion helps to alleviate the effects of dementia (Figure 13).

**Figure 14:**
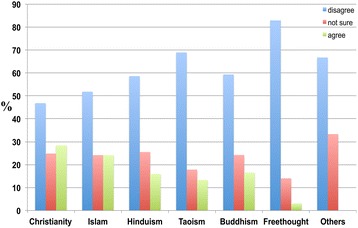
**The responses of participants from different religious backgrounds to the following statement: ‘Having a religion reduces the probability of developing dementia in the future’.** Some Christians (46.8 percent), Muslims (51.8 percent), Hindus (58.6 percent), Buddhists (59.3 percent), Freethinkers (82.9 percent), Taoists (68.9 percent) and others (66.7 percent) disagreed with the proposition that religion reduces the probability of developing dementia in the future, whereas other Christians (28.4 percent), Muslims (24.1 percent), Hindus (15.9 percent), Buddhists (16.5 percent), Freethinkers (3.1 percent), Taoists (13.3 percent) and others (0.0 percent) agreed.

The results also indicate that a significantly higher percentage of Freethinkers (82.9 percent) did not believe that religion reduces the probability of developing dementia in the future (Figure 14).

**Figure 15:**
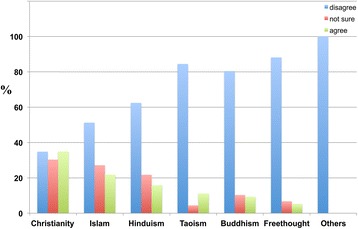
**The responses of participants from different religious backgrounds regarding whether they believed that dementia can be cured through divine healing, such as through prayer (‘Dementia can be cured through divine healing such as prayer’).** Some Christians (34.9 percent), Muslims (21.8 percent), Hindus (15.9 percent), Buddhists (9.3 percent), Freethinkers (5.2 percent), Taoists (11.1 percent) and Others (0.0 percent) agreed with the proposition, whereas other Christians (34.8 percent), Muslims (51.2 percent), Hindus (62.4 percent), Buddhists (80.4 percent), Freethinkers (88.1 percent), Taoists (84.4 percent) and Others disagreed.

Notably, respondents of all religions except Christianity disagreed with the idea that dementia could be cured by divine healing (Figure 15).

Freethinkers had the highest percentages of respondents who disagreed that religion could cure (88.1 percent), prevent (82.9 percent) or alleviate (63.2 percent) the effects of dementia and the lowest percentage of respondents who agreed that religion could cure (5.2 percent), prevent (3.1 percent) or alleviate (7.3 percent) the effects of dementia.

In contrast, it was also observed that almost the same number of Christians agreed and disagreed with the proposition that dementia can be cured by divine healing, although among all other religious groups, more participants disagreed than agreed with this proposition.

**Figure 16:**
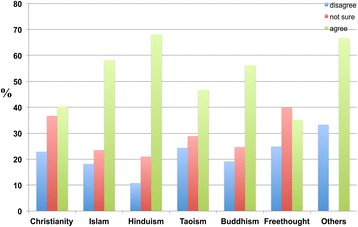
**The responses of participants to the following statement: ‘A healthy lifestyle reduces the occurrence of dementia’.** Some Christians (40.4 percent), Muslims (58.2 percent), Hindus (68.2 percent), Buddhists (56.2 percent), Freethinkers (35.2 percent), Taoists (46.7 percent), and others (66.7 percent) agreed, whereas other Christians (22.9 percent), Muslims (18.2 percent), Hindus (10.8 percent), Buddhists (19.1 percent), Freethinkers (24.9 percent), Taoists (24.4 percent), and others (33.3 percent) disagreed.

Compared to other religions, Islam, Hinduism, and Buddhism exhibited a higher percentage of respondents who agreed that a healthy lifestyle reduces or prevents the occurrence of dementia. It was also observed that relative to other religions, Hindus had the highest percentage of respondents who agreed that a healthy lifestyle reduces the occurrence of dementia. Among Christians and Freethinkers, no significant difference was observed between the numbers of respondents who agreed and disagreed (Figure 16).

### Christians have the most positive mindset regarding dementia

**Figure 17:**
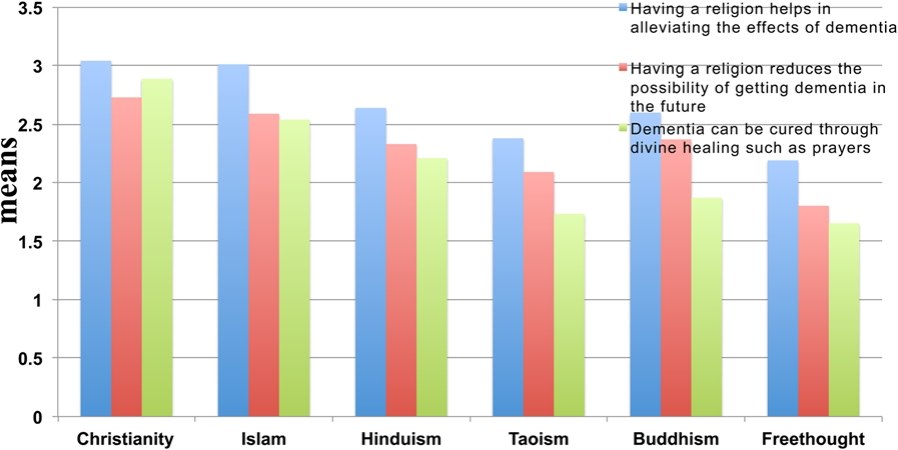
**The responses of participants to questions concerning dementia.** The responses and resulting scores ranging from 1 (strongly disagree) to 5 (strongly agree) were averaged and plotted as shown.

The participants’ responses were averaged, and the mean scores are presented in Table 3. The results indicate that, compared to other religions, Christians exhibited the most positive attitudes regarding the beneficial effects of religion on dementia. Freethinkers exhibited the least positive attitudes with respect to the potential beneficial effects of religion on dementia. Specifically, Christians held the most positive attitudes of the religious groups considered regarding whether they thought that dementia can be cured through divine healing such as prayer (Figure 17; Table 3).

**Table 3:** **Means and standard deviations of the responses of different religious groups concerning their beliefs regarding dementia**

	**Having a religionhelps to alleviatethe effects of dementia**	**Having a religionreduces theprobability ofdevelopingdementia in thefuture**	**Dementia canbe curedthrough divinehealing, suchas prayer**
Christianity	3.04 (1.040)	2.73 (1.142)	2.89 (1.180)
Islam	3.01 (1.214)	2.59 (1.180)	2.54 (1.131)
Hinduism	2.64 (1.251)	2.33 (1.201)	2.21 (1.168)
Buddhism	2.60 (1.107)	2.37 (1.118)	1.87 (1.049)
Taoism	2.38 (1.107)	2.09 (1.104)	1.73 (1.053)
Freethought	2.19 (0.913)	1.80 (0.814)	1.65 (0.859)

Standard deviations (s.d.) are presented in parentheses following the mean values.

### The effects of educational level on perceptions of aging and dementia

**Figure 18:**
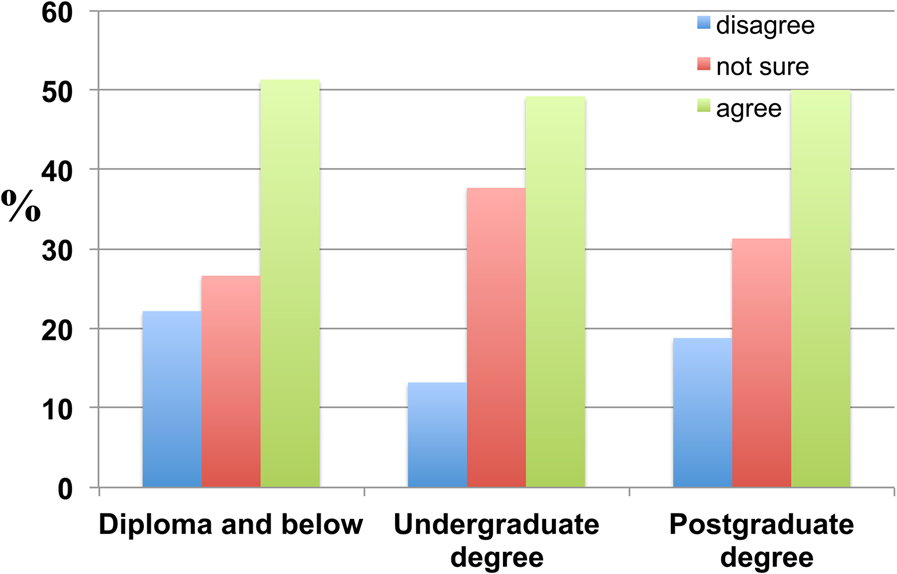
**Participants were asked whether they thought ageing was a major risk factor for dementia.** Participants were categorised according to their educational attainment: ‘Diploma and below’, ‘Undergraduate degree’ and ‘Postgraduate degree’. Of the respondents, 51.3 percent with a ‘Diploma and below’, 49.2 percent of those with an ‘Undergraduate degree’ and 50.0 percent of ‘Postgraduate degree’ holders agreed, whereas 22.2 percent of those with a ‘Diploma and below’ and 13.2 percent of ‘Undergraduate degree’ and 18.8 percent of ‘Postgraduate degree’ holders disagreed.

The results reveal that only approximately 50 percent of respondents with lower levels of education (individuals with a diploma or below and undergraduate degree holders) believed that ageing was a major risk factor for dementia. Conversely, 22.2 percent of respondents holding a diploma and below, 13.2 percent of those with undergraduate degrees and 18.8 percent of postgraduate degree holders disagreed that ageing was a major risk factor for dementia (Figure 18).

**Figure 19:**
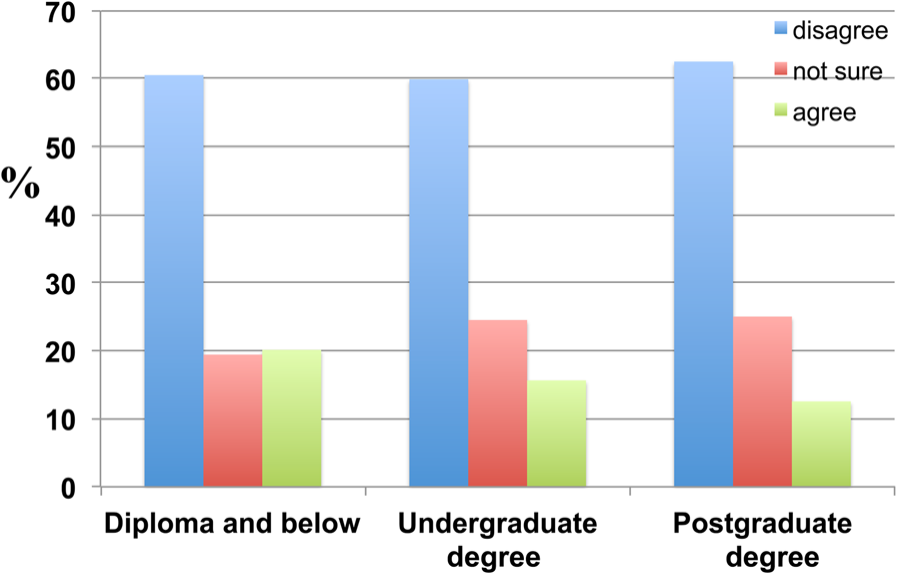
**The distribution of the education-dependent responses of the participants to the following statement: ‘Religion reduces the probability of developing dementia in the future’.** Participants with different educational levels are categorised as shown.

A significantly high percentage of individuals with a diploma or below (60.5 percent) and undergraduate degree (59.9 percent) and postgraduate degree (62.5 percent) holders disagreed with the proposition that religion reduces or prevents the occurrence of dementia (Figure 19).

**Figure 20:**
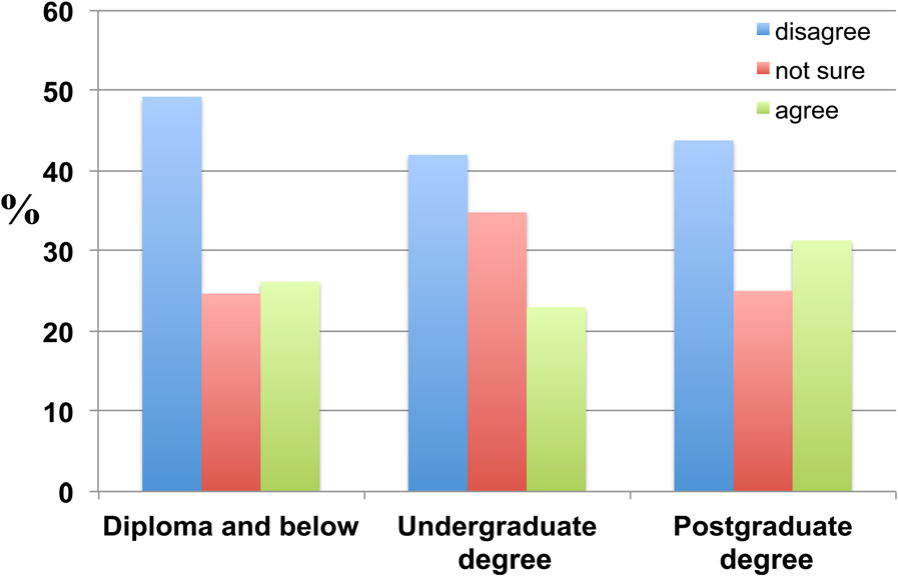
**The distribution of the education-dependent responses of participants to the following statement: ‘Having a religion alleviates the effects of dementia’.** Participants were categorised according to levels of educational attainment with respect to responses to the question of ‘disagree’, ‘not sure’ and ‘agree’.

Notably, 49.2 percent of individuals with a diploma or below and 42.0 percent of undergraduate degree and 43.8 percent of postgraduate degree holders disagreed with the proposition that having a religion helps to alleviate the effects of dementia, whereas 26.1 percent of respondents with diploma and below and 23.0 percent of undergraduate degree and 31.3 percent of postgraduate degree holders agreed. There was no significant difference between the numbers of respondents who agreed and disagreed, and a higher percentage of individuals with a diploma or below agreed that religion alleviates the effects of dementia than undergraduate and postgraduate degree holders (Figure 20).

**Figure 21:**
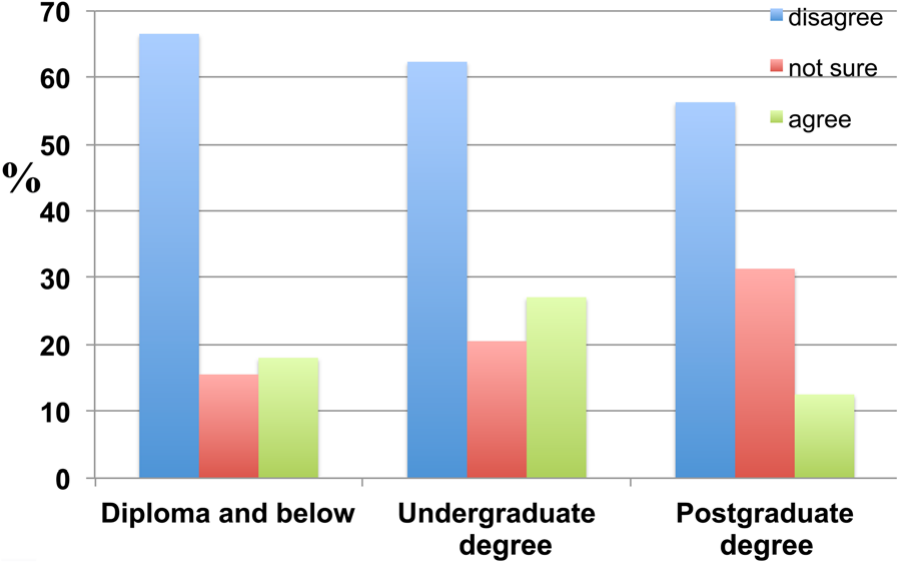
**This figure depicts the responses of participants to whether dementia can be cured by divine healing through prayer (‘Dementia can be cured by divine healing, such as through prayer’).** The education level-dependent responses of participants are categorised according to educational attainment: ‘Diploma and below’, ‘Undergraduate degrees’ and ‘Postgraduate degrees’. ‘Strongly disagree’ and ‘agree’ responses were combined into a single subscale of ‘disagree’. ‘Strongly agree’ and ‘agree’ responses were combined into a single subscale of ‘agree’. The responses were as follows: 66.5 percent of individuals with a ‘Diploma and below’ and 62.3 percent of ‘Undergraduate degree’ and 56.3 percent of ‘Postgraduate degree’ holders do not believe that divine intervention is able to cure dementia.

However, the majority does not believe that divine intervention is able to cure dementia (Figure 21).

A higher percentage of respondents with a diploma or below disagreed that divine healing can cure dementia compared to undergraduate and postgraduate degree holders.

**Figure 22:**
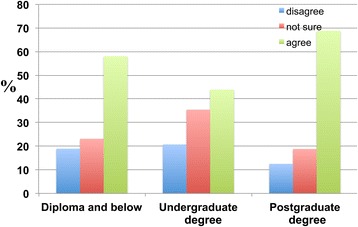
**The education-dependent responses of participants regarding whether a healthy lifestyle reduces the occurrence of dementia.** Participants were categorised according to their level of educational attainment. The ‘Disagree’ bar represents the combination of the subscales ‘strongly disagree’ and ‘disagree’. The ‘Agree’ bar represents the combination of the subscales ‘strongly agree’ and ‘agree’. Affirmative responses were reported by 58.0 percent of individuals with a diploma or below and 43.9 percent of undergraduate degree and 68.8 percent of postgraduate degree holders.

Across all educational attainment categories, the majority agreed with the proposition that a healthy lifestyle would reduce the occurrence of dementia (Figure 22).

The difference between undergraduate degree holders who disagreed and agreed was smaller than the differences between responses for those with a diploma or below and holders of postgraduate degrees. A significant number of postgraduate degree holders (68.8 percent) agreed that a healthy lifestyle reduces the occurrence of dementia.

### Attitudes and perceptions of different Age groups regarding ageing and dementia

It was hypothesised that as age increases, individuals would be more likely to have an improved knowledge and understanding of dementia. Age was categorised into five groups because it was thought that there might be a significant difference in the responses between these age groups. The results of a correlation analysis indicated that age was significantly correlated with participants’ responses to the questions (Table 4).

**Table 4:** **Correlations of age with participant responses to statements regarding ageing and dementia**

	**Age**	
	**Correlation coefficients (** ***n*** **=980)**	***P*** **-value (two-tailed)**
Have heard of dementia.	0.116**	.000
Ageing is a major risk factor for dementia.	0.070**	.029
People with dementia should be placed in homes.	0.175**	.000
People with severe dementia should becared for by professionals instead of familymembers.	0.190**	.000

**p<0.01.

There is a clear relationship between age and individuals’ perceptions and attitudes regarding seeking help for ageing and dementia (Table 5).

**Table 5:** **The responses of participants from different participant age groups to statements concerning ageing and dementia**

		**Age groups in years**			
	19-30	31-40	41-55	56-65	>65^a^
	(*n*=655)	(*n*=100)	(*n*=104)	(*n*=106)	(*n*=15)
	%	%	%	%	%
**I have heard of dementia**					
Definitely have not heard of it.	6.4	18.0	10.6	5.7	0.0
May have heard of it.	5.8	6.0	6.7	3.8	0.0
Not sure.	18.8	14.0	10.6	1.9	6.7
Somewhat aware of it.	49.5	39.0	52.9	35.8	73.3
Definitely aware of it.	19.5	23.0	19.2	52.8	20.0
**Ageing is a major risk factor for dementia**					
Strongly disagree	3.1	3.0	7.7	9.4	33.3
Disagree	11.0	20.0	15.4	12.3	13.3
Not sure	40.2	32.0	14.4	7.5	13.3
Agree	33.6	29.0	47.1	41.5	33.3
Strongly agree	12.2	16.0	15.4	29.2	6.7
**People with dementia should be placed in homes**					
Strongly disagree	23.8	21.0	24.0	29.2	33.3
Disagree	49.3	39.0	39.4	31.1	26.7
Not sure	20.0	21.0	11.5	10.4	0.0
Agree	5.3	10.0	12.5	15.1	13.3
Strongly agree	1.5	9.0	12.5	15.1	26.7
**People with severe dementia should be cared for by professionals instead of family members**					
Strongly disagree	8.7	8.0	9.6	18.9	13.3
Disagree	37.9	25.0	26.0	14.2	13.3
Not sure	28.1	25.0	14.4	6.6	6.7
Agree	19.1	32.0	38.5	35.8	40.0
Strongly agree	6.3	10.0	11.5	24.5	26.7

^a^Results for this group should be interpreted with caution because the sample size was small.

**Figure 23:**
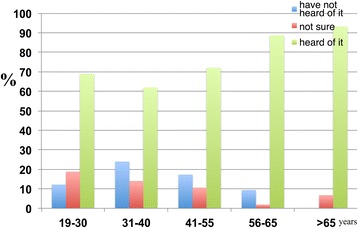
**The age-dependent responses of participants to the following statement: ‘I have heard of dementia’.** The numbers of participants who ‘have not heard of it’, ‘not sure’ and ‘heard of it’ were calculated as percentages. It was found that 69.0 percent of the 19–30 age group, 62.0 percent of the 31–40 age group, 72.0 percent of the 41–55 age group, 88.7 percent of the 56–65 age group and 93.3 percent of the >65 age group were aware of dementia.

The results also indicate that higher percentages of respondents in the 41–55, 56–65 and >65 age groups were aware of dementia than those in the 19–30 and 31–40 age groups (Figure 23).

**Figure 24:**
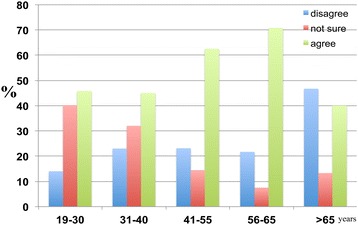
**The age-dependent responses of participants to the following statement: ‘Ageing is a major risk factor for dementia’.** The responses of participants were categorised into ‘disagree’, ‘not sure’ and ‘agree’. The results were as follows: 45.8 percent of the 19–30, 45.0 percent of the 31–40, 62.5 percent of the 41–55, 70.8 percent of the 56–65 and 40 percent of the >65 age groups thought that ageing was a major risk factor for dementia.

With respect to risk factors, there are relatively higher percentages of respondents in the 41–55 and 56–65 age groups who considered ageing to be a major risk factor for dementia (Figure 24).

**Figure 25:**
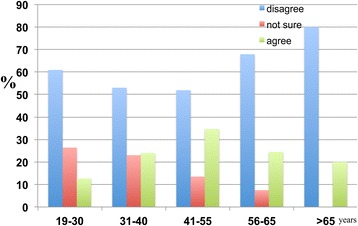
**The age-dependent responses of participants to the following statement: ‘Having a religion reduces the probability of developing dementia in the future’.** The responses were as follows: 60.9 percent of the 19–30 age group, 53.0 percent of the 31–40 age group, 51.9 percent of the 41–55 age group, 67.9 percent of the 56–65 age group and 80.0 percent of the >65 age group disagreed with the proposition.

Across all age groups, the majority clearly disagreed with the proposition that religion was able to reduce or prevent the occurrence of dementia. As age increased, the number of respondents who disagreed with the proposition that religion prevents dementia also increased (Figure 25).

**Figure 26:**
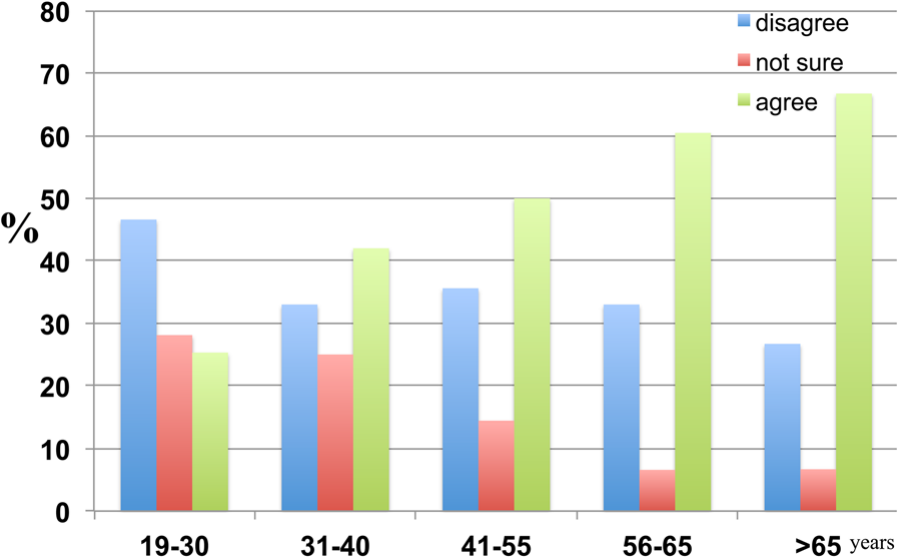
**The responses of participants to the following statement: ‘Dementia patients should be cared for by professionals instead of family members’.** With 46.6 percent of the 19–30 age group, 33.0 percent of the 31–40 age group, 35.6 percent of the 41–55 age group, 33.0 percent of the 56–65 age group and 26.7 percent of the >65 age group disagreeing, and with 25.3 percent of the 19–30 age group, 42.0 percent of the 31–40 age group, 50.0 percent of the 41–55 age group, 60.4 percent of the 56–65 age group, and 66.7 percent of the >65 age group agreeing with this proposition.

The results also reveal a gradual, age-dependent decrease in the percentage of participants who disagree with the notion of hiring health care professionals to care for dementia patients. There was a corresponding age-dependent increase in the percentage of respondents who agree with the notion of hiring professionals to care for dementia patients (Figure 26).

**Figure 27:**
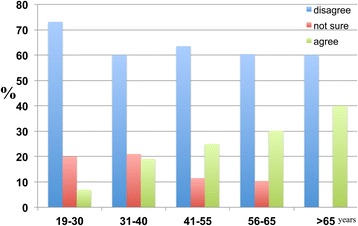
**The age-dependent percentage responses of participants to the following statement: ‘Dementia patients should be placed in homes.** 73.1 percent of the 19–30 age group, 60.0 percent of the 31–40 age group, 63.5 percent of the 41–55 age group, 60.4 percent of the 56–65 age group and 60.0 percent of the >65 age group disagreed with the notion, whereas 6.9 percent of the 19–30 age group, 19.0 percent of the 31–40 age group, 25.0 percent of the 41–55 age group, 30.2 percent of the 56–65 age group, and 40.0 percent of the >65 age group agreed with this proposition.

Independent of age, the majority disagreed with the notion of placing dementia patients in homes. As age increased, the percentage of respondents who agreed with placing dementia patients in homes also increased (Figure 27).

### Relationship between multiple variables regarding ageing and dementia

The purpose of the multiple regression analyses performed in this study was to determine the degree to which each factor influences perceptions of ageing and dementia while accounting for other factors.

The results indicate that educational levels (B=0.057, t(980)=2.702, p<.01) and religion (B=−0.050, t(980)=−7.906, p<.001) were statistically significant in influencing participants’ responses when asked whether religion alleviates the effects of dementia (Table 6). A positive beta value indicates a positive relationship between educational attainment and whether respondents agree that religion alleviates the effects of dementia.

**Table 6:** **The results of a multiple regression analysis based on age, gender, ethnicity, religion, and education of a random sample of 980 respondents**

	**Having a religion helps to alleviate the effects of dementia**			**Having a religion reduces the probability of developing dementia in the future**		
	**B (Unstandardised coefficient)**	**β (Standardised coefficient)**	***t*** **-statistic**	***B***	**β**	***t***
Age	0.033	0.033	0.824	0.022	0.022	0.549
Gender	−0.056	−0.024	−0.740	−0.095	−0.041	−1.274
Ethnic	0.017	0.012	0.374	−0.010	−0.008	−0.231
Religion	−0.050	−0.256	−7.906***	−0.054	−0.278	−8.632***
Education	0.057	0.107	2.702**	0.026	0.050	1.263
	Multiple R=0.269			Multiple R=0.283		
	R^2^=0.072			R^2^=0.080		
	Adjusted R square=0.067			Adjusted R square=0.075		
	F=14.724***			F=16.456***		

The relationship between respondent age, gender, ethnicity, religion, and education, on the one hand, and participants' responses to each statement, on the other, is tested.

**p<.01; ***p<.001.

Religion was a statistically significant factor in predicting responses to the following statement: ‘Having a religion reduces the probability of developing dementia in the future’. Religion (B=−0.054, t(980)=−8.632, p<.001) was significant as a predictor of the response to this statement (Table 6).

With respect to the following statement: ‘Dementia can be cured through divine healing, such as through prayer’, religion (B=−0.057, t(980)=−8.938, p<.001) was significant as a predictor of the participants’ responses to this question (Table 7).

**Table 7:** **The results of a multiple regression analysis based on age, gender, ethnicity, religion, and education of a random sample of 980 respondents**

	**Dementia can be cured through divine healing, such as through prayer**			**People with dementia should be placed in ‘homes’**		
	***B***	**β**	***t***	***B***	**β**	***t***
Age	0.013	0.013	0.333	0.134	0.141	3.495***
Gender	−0.029	−0.012	−0.377	−0.024	−0.011	−0.328
Ethnic	0.069	0.051	1.546	0.022	0.018	0.520
Religion	−0.057	−0.286	−8.938***	−0.002	−0.012	−0.377
Education	0.034	0.063	1.610	−0.029	−0.058	−1.427
	Multiple R=0.301			Multiple R=0.186		
	R^2^=0.091			R^2^=0.034		
	Adjusted R square=0.086			Adjusted R square=0.029		
	F=18.810***			F=6.730***		

The relationship between respondent age, gender, ethnicity, religion, and education, on the one hand, and participants’ responses to each statement, on the other, is tested.

***p<.001.

When asked about placing patients with dementia in homes, the results indicate that age was statistically significant in predicting responses (Table 7; Figure 27). The multiple regression analysis revealed that age was related to individuals’ attitudes and perceptions with respect to seeking help for dementia. Most respondents in the 19–30 age group did not wish to place their elderly or ill relatives in homes and would prefer to co-reside with their parents. Notably, more respondents in the older age groups (41–55 and 56–65 versus 19–30 and 31–40) wished to place the elderly in homes or hire professionals to care for them instead of placing the burden on family members. The positive beta value (B=0.134) indicates a positive relationship between age and participants’ responses.

Age, religion, and education were statistically significant in predicting participants’ responses to the following statement: ‘Dementia is related to ageing’ (Table 8).

**Table 8:** **The results of a multiple regression analysis based on age, gender, ethnicity, religion, and education of a random sample of 980 respondents**

	**Dementia is related to ageing**			**People with severe dementia should be cared for by professionals instead of by family members**		
	***B***	**β**	***t***	***B***	**β**	***t***
Age	0.118	0.128	3.196***	0.201	0.197	4.913***
Gender	0.049	0.04	1.184	0.039	0.017	0.506
Ethnic	−0.1	10.56	−1.687	0.108	0.080	2.378**
Religion	0.321	0.151	4.594***	−0.006	−0.032	−0.975
Education	0.071	0.146	3.613***	0.010	0.020	0.486
	Multiple R=0.195			Multiple R=0.214		
	R^2^=0.038			R^2^=0.046		
	Adjusted R square=.033			Adjusted R square=0.041		
	F=7.473***			F=9.082***		

The relationship between respondent age, gender, ethnicity, religion, and education, as well as the participants' responses to each statement, is tested.

**p<.01; ***p<.001.

The multiple regression analysis demonstrated that age, religion, and level of educational attainment were significantly correlated with knowledge of dementia. The positive beta values indicate a positive relationship between knowledge of dementia and either i) age, ii) religion, or iii) an individual’s level of educational attainment. Interestingly, only respondents with higher levels of education believed that dementia was related to ageing.

When asked about hiring professionals to care for patients with dementia, age was also a significant predictor of responses. A positive beta value (B=0.201) suggests a positive relationship between age and participants’ responses to the statement.

## Discussion

The aim of the current investigation was to explore the various contexts in which the debate concerning ageing and dementia occurs and examine how the threat is perceived, particularly by younger generations, in different social and religious communities in a multi-cultural city such as Singapore. Using a social theory lens, the study focused not only on cultural and contextual practices but also on epistemological orientations in education concerning ageing and dementia that shape communities, societies, and cultures, in addition to individual interpretations of this problem.

Ageing is one of the major risk factors for dementia (Mucke 2009; Manavalan et al. 2013). Scientific research has been undertaken to determine the causes of and treatments for dementia at the molecular and cellular level, but relatively little attention has been devoted to caregiving (Quinn et al. 2009). Researchers believe that educational, cultural, religious, and ethnic backgrounds may affect the caregiving and coping strategies pursued for dementia patients (Montgomery and Williams 2001). Because societies across the globe are ageing, it is important that governments inform younger generations about ageing and dementia. This investigation thus focused on the perception of younger individuals in Singapore, a multi-cultural city.

### Attitudes and perceptions from different ethnic groups regarding ageing and dementia

The discrepancy between awareness of dementia and recognition that ageing could be a major risk factor for dementia indicates that individuals might have become aware of dementia without understanding its causes. Because cancer is the leading cause of death in Singapore, more individuals are familiar with cancer than dementia (Singapore Cancer Registry, 2008). Dementia has only recently gained recognition as the population ages and as individuals gradually become more aware of the risk factors (Sahadevan et al. 1999; Singapore Ministry of Social and Family Development 2009).

In Singapore, annual campaigns are organised to increase awareness of the need to live a healthy lifestyle (Singapore Government Health Promotion Board 2013). More respondents in the Chinese, Malaysian, Indian, and Pakistani ethnic groups agreed that a healthy lifestyle reduces the occurrence of dementia. There are no significant differences in attitudes and perceptions regarding ageing and dementia among different ethnic groups, most likely because lifestyles have increasingly conformed over many years, becoming similar across the Singaporean population. However, in the USA and Western Europe, which have much larger populations, different nations, states or provinces have different cultures (ten Have et al. 2010; Rosenbaum 1989; Sheikh and Furnham 2000).

### Attitudes and perceptions from different religious backgrounds regarding ageing and dementia

Among followers of Islam, Hinduism, and Buddhism, a higher percentage of respondents agreed with the statement that a healthy lifestyle reduces or prevents the occurrence of dementia. This is likely the result of habits developed during the practice of their faiths; some religions advocate healthy living and eating habits. Certain types of food may be avoided because of religious prohibitions or the potential harm to the body. For example, Hindus practice yoga to maintain a healthy state of mind and body.

While statistically equivalent shares of Christians disagreed and agreed that religion was able to cure, prevent or alleviate the effects of dementia, among all religions, Christians seem to have the most positive attitudes toward dementia. This result might be explained by widespread eyewitness accounts, personal experiences or evidence of miracle healings reported worldwide.

As a Freethinker is defined as an individual who forms opinions based on reason and doubts religious dogma, this explains why a higher percentage of Freethinkers disagreed with statements concerning the beneficial effects of religion on ageing and ageing-related dementia.

Research has shown that religion or spirituality generates positive bio-psychosocial outcomes because of strong social support in the community and greater optimism (Ackerman et al. 2009; Ferraro and Albrecht-Jensen 1991; Idler 1987; Mitchell and Weatherly 2000; Vance et al. 2008). This may explain why the number of respondents in this study who disagreed with the statement that religion alleviates the effects of dementia was much smaller than the number who disagreed with the statement that divine healing can cure dementia. Despite being religious, most of the participants did not believe in miracles or divine healing, perhaps because they thought it was scientifically impossible. Nevertheless, individuals continue to participate in religious activities because of the relationships and support provided in the community. Other potential reasons include the desire to find meaning and a purpose in life or to find hope and strength to cope with circumstances in life or chronic illnesses (Chandler et al. 1992; Chappelle 2002; Espeland 1999; Harmon et al. 1985; O’Brien 1999; Sawatzky et al. 2005). For some, religion is an intergenerational tradition that individuals follow as a means of connecting with the practices of their parents or for solely legalistic reasons. However, there are some religious individuals who truly believe in miracles and that prayer can heal illnesses such as dementia.

### Educational level and perceptions of ageing and dementia

As Singapore industrialises, more Singaporeans are receiving better education and completing higher levels of it. Typically, the concepts or theories taught in schools oppose or disprove beliefs in supernatural phenomena (Bowles and Gintis 1976). Although some Singaporeans only receive primary or secondary education, the internet, other media resources, and information are readily available to the public. Singapore is industrialised and developed to the extent that, regardless of educational attainment, Singaporeans look to science to explain diseases. Research by Lee and Bishop (2001) has shown that psychological explanations were significant for less-educated individuals.

It can be assumed that with higher levels of education, individuals would be less likely to believe in miracles or supernatural phenomena and would have a better understanding of dementia (Glaeser and Sacerdote 2002; Mahner and Bunge 1996). Indeed, the present results reveal that the level of educational attainment was significantly correlated with understanding dementia and that more respondents with higher levels of education believed that dementia was related to ageing.

However, extensive research on the relationship between education and religion has revealed that the level of educational attainment is negatively correlated with religious beliefs (Bertram-Toost et al. 2007; Glaeser and Sacerdote 2002; Rieff 1996; Swatos and Christiano 1999). Surprisingly, the results presented here reveal that respondents with lower levels of education (those with diplomas or below) were less likely to believe that religion can alleviate the effects of dementia than undergraduate or postgraduate degree holders. In addition, a significantly higher percentage of participants in all three educational groups did not believe in divine healing. This finding most likely indicates that, independently of educational attainment, individuals generally believe that religion improves one’s ability to cope with dementia but do not believe that religion helps to prevent or cure it.

### Attitudes and perceptions of different Age groups regarding ageing and dementia

In the present analysis, the findings reveal that as people age, they are more likely to agree with the proposition that dementia is related to ageing or that ageing is a major risk factor for dementia. This finding suggests that individuals improve their understandings and knowledge of ageing and dementia as they age owing to the risk of developing age-related diseases (Ayalon and Areán 2004). It also indicates that the government must make younger generations more aware of ageing-related problems to encourage them to act responsibly early in life – not only with respect to their own lifestyles but also with respect to care-taking.

### Ageing and care-taking

A higher percentage across all ethnic and age groups disagreed with the notion of placing elderly patients with dementia in specialised care-taking centres (homes), a pattern of response that may reflect Singaporean beliefs regarding intergenerational support. Another potential explanation is financial, as the expenses of placing dementia sufferers in homes for the elderly are high (Knodel and Nibhon 1997). However, a higher percentage of participants in all ethnic groups reported that they preferred to hire professionals to care for dementia patients rather than place them in homes. Research by Heok and Li (1997) indicated that the reduced availability of female caregivers and the increased number of smaller families has increased the need to seek help from outside the family, and the number of elderly living alone or in homes is increasing. The observation that people in the older age groups wished to place the elderly in homes or hire professionals to care for them instead of placing the burden on family members may reflect smaller family sizes in Singapore, the increased number of Singaporeans who work (which may also have reduced time available to care for family members with dementia) and the fact that respondents in these age groups are the primary caregivers for the elderly. Therefore, hiring a professional meets the special needs of the elderly and enables their children to remain by their side. The stress of caregiving could result from insufficient knowledge regarding dementia, poor coping strategies, a lack of time due to job responsibilities and a lack of social or familial support. Moreover, no financial assistance to place elderly or ill individuals in homes is provided by the government, as Singaporean government policies encourage the co-residence of elderly parents and their children.

As Singapore becomes more industrialised, and Singaporeans become wealthier, the social function of the family and of co-residence may decline. In the USA and western Europe, multigenerational co-residence is very rare, which is most likely a result of industrialisation (Hollinger and Haller 1990; Silverstein et al. 1998).

### Limitations of the current study

As with all surveys, limitations must be considered. While the total number of participants can be enlarged by up to three times, the individual sub-groups within each parameter (e.g., age, ethnicity, and religion) would need to be more equally represented. Depending on available resources, the applied questionnaire could be extended by adding additional detailed questions to obtain more convincing and definitive conclusions. Such issues must be addressed in future analyses.

## Conclusions

### Directions for future research

This study considered respondents’ awareness of ageing and dementia from a cross-cultural epistemological perspective to determine how individuals’ perceptions are influenced by their religious backgrounds, culture, age, and level of educational attainment; in particular, the study focused on younger generations as they prepare for ageing-related problems in the future. These factors work interchangeably to influence individual perceptions and attitudes. The perceptions and needs of older individuals regarding life differ from those of younger individuals. By determining how these factors influence lifestyles and perceptions pertaining to ageing and dementia, we can develop appropriate strategies to cope with and care for dementia patients. Although the present pilot study provides valuable insights into the younger generation’s perspective across a wide range of cultural and religious viewpoints, this cross-sectional study does not necessarily permit a final, definitive interpretation. Accordingly, a subsequent longitudinal study would be required to determine whether ethnicity, age, religion, and levels of educational attainment are significant factors that influence an individual’s perceptions at different points of the course of life.

With such plentiful scientific research on dementia currently underway, ageing and dementia should be examined from a socio-cultural perspective in more detail. Thus, it would be of interest to extend the current pilot study to investigate an even larger cohort across each variable (e.g., age, ethnicity, and religion). Ultimately, such a study should be conducted and compared with investigations in other cities around the world, for example, Berlin, London, Hong Kong, Seoul, New York, and Sydney.

**Figure 28:**
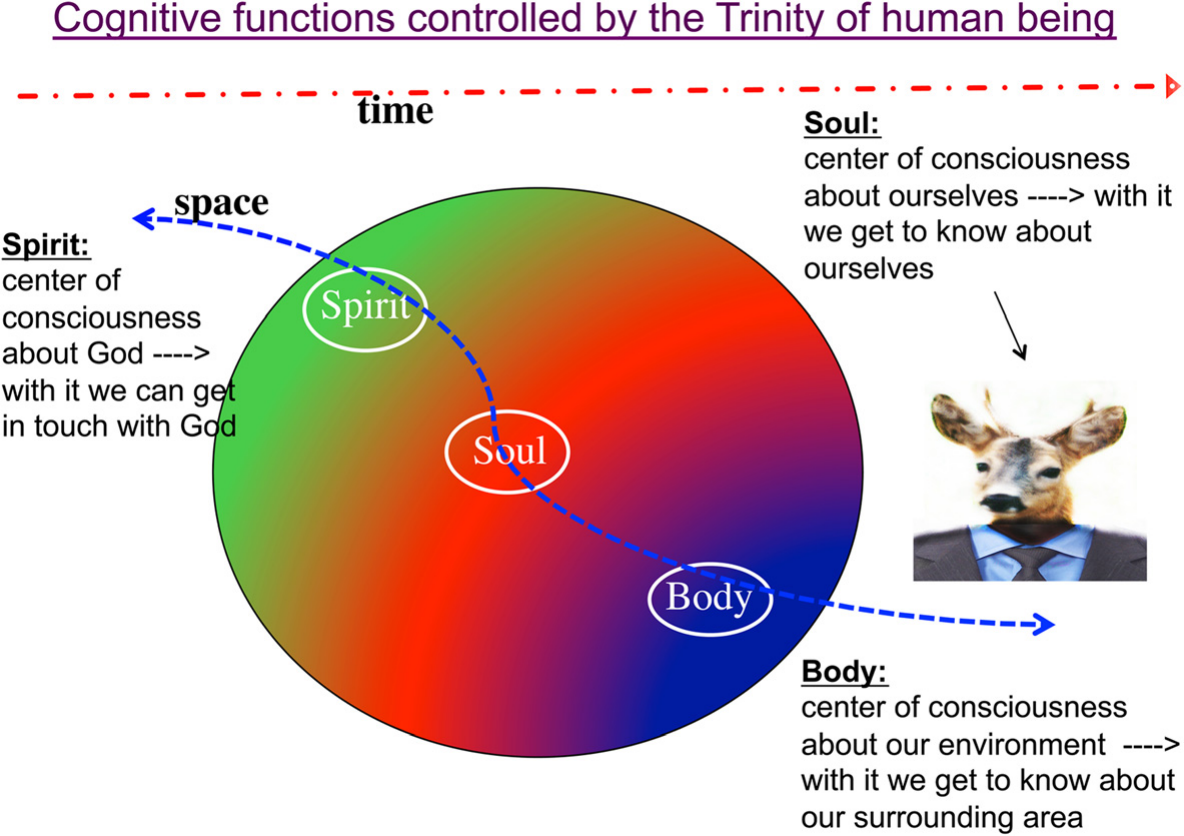
**Human beings’ cognitive functions and mindsets are controlled by various objective and subjective parameters that must be considered when evaluating human behaviour.**

Cures for dementia have yet to be found, and research must be conducted to identify the types of lifestyles that will benefit the population as a whole. Religious individuals premise the meaning and purpose of their lives on a transcendent relationship with a being superior to themselves. As such, hope and strength come not from within but from a transcendent being. Faith in a superior being not of this world may provide the hope and volition necessary to continue living despite unfavourable and harsh circumstances. Consequently, assuming that human beings’ cognitive functions and mindsets are controlled by the trinity, an individual’s decisions regarding, for example, lifestyle may depend not only on his subjective belief concerning the existence of such a trinity. However, the ultimate decision may further depend on an individual’s beliefs regarding human existence in terms of time, space, and destiny itself, which, in turn, can be affected by various other parameters such as ageing, education, social, and cultural environment, etc. (Figure 28).

Extensive research into transcultural perspectives on mental illnesses demonstrates that cultural and religious groups have varying perspectives on mental illness (Draguns 1984; Marsella and White 1982; Sheikh and Furnham 2000). Religion and culture interact interchangeably and influence individuals’ perceptions and behaviours regarding ageing and ageing-related dementia. While many do not believe that religion can prevent, alleviate or cure dementia, individuals continue to engage in religious activities because they enhance their quality of life through communal relationships and other social benefits. As the population ages, a larger proportion of the population may suffer from disease, and it will become imperative for younger generations across all cultural and religious groups to increase their awareness of ageing-related illnesses, such as dementia. This will not only allow the population to develop healthy lifestyles to prevent dementia in the future, it will also help develop coping strategies to provide quality care for the elderly.

### Ethics statement

All measurements have been carried out on human being complying with local laws.
